# Prediction of Metabolic Flux Distribution from Gene Expression Data Based on the Flux Minimization Principle

**DOI:** 10.1371/journal.pone.0112524

**Published:** 2014-11-14

**Authors:** Hyun-Seob Song, Jaques Reifman, Anders Wallqvist

**Affiliations:** Department of Defense Biotechnology High Performance Computing Software Applications Institute, Telemedicine and Advanced Technology Research Center, U.S. Army Medical Research and Materiel Command, Fort Detrick, Maryland, United States of America; Universidad de La Laguna, Spain

## Abstract

Prediction of possible flux distributions in a metabolic network provides detailed phenotypic information that links metabolism to cellular physiology. To estimate metabolic steady-state fluxes, the most common approach is to solve a set of macroscopic mass balance equations subjected to stoichiometric constraints while attempting to optimize an assumed optimal objective function. This assumption is justifiable in specific cases but may be invalid when tested across different conditions, cell populations, or other organisms. With an aim to providing a more consistent and reliable prediction of flux distributions over a wide range of conditions, in this article we propose a framework that uses the flux minimization principle to predict active metabolic pathways from mRNA expression data. The proposed algorithm minimizes a weighted sum of flux magnitudes, while biomass production can be bounded to fit an ample range from very low to very high values according to the analyzed context. We have formulated the flux weights as a function of the corresponding enzyme reaction's gene expression value, enabling the creation of context-specific fluxes based on a generic metabolic network. In case studies of wild-type *Saccharomyces cerevisiae*, and wild-type and mutant *Escherichia coli* strains, our method achieved high prediction accuracy, as gauged by correlation coefficients and sums of squared error, with respect to the experimentally measured values. In contrast to other approaches, our method was able to provide quantitative predictions for both model organisms under a variety of conditions. Our approach requires no prior knowledge or assumption of a context-specific metabolic functionality and does not require trial-and-error parameter adjustments. Thus, our framework is of general applicability for modeling the transcription-dependent metabolism of bacteria and yeasts.

## Introduction

Cellular metabolism involves a myriad of regulatory processes and metabolic components functioning together through a complex set of interactions and reactions. Although “omics” technologies provide an increasingly large body of information on each individual component involved in metabolism, our knowledge of how these components as a system give rise to multiple phenotypes under different conditions is far from complete. A powerful approach to investigate metabolism and metabolic processes is to analyze the flow of material and energy through a metabolic network. In particular, the analysis of metabolite fluxes in a metabolic network serves as an essential tool in many biotechnology and biomedical applications, for example, to enhance the production of food and biofuels [1], identify disease biomarkers and drug targets [2], [3], and study complex human physiological processes [4]. Metabolite flows in a network can be determined by experimental or computational techniques.

A standard experimental technique to quantify the distribution of fluxes in a network is to perform a metabolic flux analysis (MFA), which is based on isotope labeling techniques (mostly using ^13^C) [5]. ^13^C-MFA traces isotope-labeled metabolites using mass spectrometry and determines individual reaction fluxes by fitting ^13^C data to a network model with the help of additional measurements on exchange fluxes, such as nutrient uptake and product excretion rates. Due to experimental difficulties in obtaining quantitative and precise measurements that cover a large-size network with diverse pathways and many metabolites, the use of ^13^C-MFA is typically limited to the determination of fluxes related to the central carbon metabolism [6]. The most common computational techniques used for the analysis of genome-scale networks are flux balance analysis (FBA) and its derivatives [7], [8]. FBA postulates steady-state cellular metabolism as being driven toward maximizing a certain fitness function (typically, biomass production) and estimates the flux distribution by solving a linear programming (LP) problem. Modification of the FBA algorithm to incorporate additional biological information from gene expression profiles is often used to generate context-dependent flux estimates for specific biological conditions without changing the fundamental optimization criterion of the algorithm.

Although gene transcripts are not a direct readout of enzyme activities, as posttranscriptional events determine cellular protein concentrations and activity, a number of applications have shown that they provide important cues for the likelihood that associated reactions are activated [9]–[13]. These studies include the pioneering work of Shlomi et al. [14], who identified distinct metabolic activity in 10 different human cancer tissues. Our previous work in this area includes the prediction of metabolic adaptation of *Mycobacterium tuberculosis* under hypoxic and anaerobic conditions [15] and the development of a kinetic modeling framework to predict phenotypic alterations of *Saccharomyces cerevisiae* in response to chemical treatments [16]. Depending on the experimental design and platform, gene transcriptional expression profiles are collected either as absolute or differential values. Metabolic network integration algorithms that depend on differential expression data generally require reliable measurements or estimates of the flux distribution at a reference condition. The availability of a well-characterized biological reference state provides a robust starting point for investigating perturbed states or conditions. However, data for a reference state may not always be available or even obtainable. Absolute gene expression data are more typically used when studying complex tissues, where a reference no longer strictly refers to a unique cell population or metabolic state. Our interest lies in generating a generalized methodology that can use absolute gene transcriptional data with a minimum number of assumptions, constraints, parameters, or auxiliary data inputs.

Existing methods that similarly address these issues using absolute expression data include E-Flux [17], integrative metabolic analysis tool (iMAT) [14], [18], and the algorithm developed by Lee et al. [19]. These methods vary in their assumptions and the approaches used to constrain the solutions of the metabolic fluxes based on gene transcription data. E-Flux can be regarded as an extended version of FBA with additional flux constraints, i.e., it maximizes biomass yield under the imposed upper and lower limits on fluxes determined as a function of their associated gene expression levels. In contrast, iMAT maximizes the fit between gene expression and the flux state of the network such that the number of reactions that are highly expressed and carry a flux is maximized, while the number of reactions that are under-expressed and carry a flux is minimized. Finally, the algorithm recently presented by Lee et al. maximizes the correlation between flux magnitudes and the associated gene expression levels. Importantly, none of these methods have an explicit requirement for defining a cellular metabolic object through an optimal biomass production. Although maximization of biomass production as used in E-Flux and FBA has been exploited to great advantage in many simulations and analyses of microbial growth, the general assumption is context- and growth-condition specific [20], [21] and is not supported for multicellular organisms and tissues [19], [22]. While other alternative objective functions, such as maximizing or maintaining a fixed level of ATP production or other vital cellular component, could be considered, it is difficult to know *a priori* which one is the most appropriate in a given condition [23], [24].

The methods discussed above have been shown to provide accurate and detailed predictions of flux distributions for specific systems, however, as shown in this article, they do not perform consistently across different conditions and organisms. Thus, our aim was to create a more broadly applicable computational approach that does not heavily rely on context-specific knowledge and assumptions. Our approach is based on flux minimization, with the hypothesis that flux magnitudes are proportional to enzyme concentrations, and that cells are frugal in synthesizing enzymes due to limited internal resources (such as ribosomes, RNA polymerases, and ATP) [21], [25]. The principle of flux minimization has been used successfully to estimate the metabolic states (i.e., flux distributions) in uncharacterized environments in EXploration of Alternative Metabolic Optima (EXAMO) [26]. As a preceding step, the implementation of EXAMO requires the reconstruction of an environment-specific subnetwork, which generally includes a number of iterative curation procedures and heuristic decisions. In contrast, our algorithm predicts flux distributions from a generic network by minimizing a weighted sum of flux magnitudes, where the weights are a function of the corresponding gene expression levels. A similar idea was used in Gene Inactivity Moderated by Metabolism and Expression (GIMME) [27], which is a framework for assembling context-specific networks from a large set of metabolic reactions, but not aimed at predicting flux distributions. In this work, we developed an LP-based framework by modifying GIMME for quantitative prediction of flux distributions. Whereas GIMME requires certain metabolic functionalities to be active above condition-dependent thresholds, we removed these decisions by forcing biomass production to carry nonzero flux. In both GIMME and our algorithm, the use of absolute gene expression data provides the ability to use transcriptional data taken from a single experimental condition where a control condition may not be available. We have termed our method “expression data-guided flux minimization” (E-Fmin).

We evaluated the developed E-Fmin algorithm through case studies of *S. cerevisiae* and *Escherichia coli* metabolism by a comparison with experimentally determined flux data and other model predictions discussed above. The analysis showed that, whereas other algorithms performed well for one condition/organism, our algorithm showed consistently good predictions of flux distribution for both organisms under different conditions. Thus, we believe that E-Fmin will provide a robust capability for analyzing complex metabolic systems.

## Results and Discussion

### Structure of the E-Fmin Algorithm

E-Fmin employed a similar structure to that of GIMME, with the following optimization problem that minimizes a weighted sum of flux magnitudes, i.e., 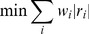
(1)subject to



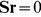
(2)

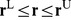
(3)

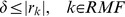
(4)where *r_i_* is the *i* ^th^ flux, its weight *w_i_* is a function of gene expression level, 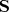 is the (*m*×*n*) stoichiometric matrix, **r** is the vector of 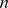 fluxes, **r** ^L^ and **r** ^U^ are vectors of upper and lower limits of **r**, respectively, RMF stands for required metabolic functionalities, and 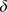 is a threshold to be specified as an input. *Eq. 2* denotes stoichiometric balances of fluxes under steady-state conditions. Flux bounds in *Eq. 3* are determined by accounting for their directionality as constrained by thermodynamics, i.e., upper and lower bounds are ±∞ if a reaction is reversible, and if one of the reactions is irreversible, the opposite reaction bound was set to zero. By the inequality constraint in *Eq. 4*, one or more reactions classified as RMF are forced to carry fluxes above 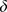. GIMME formulates the weight *w_i_* as 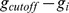, if 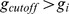, and as 0 otherwise, where 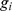 is the gene expression level mapped on the *i* ^th^ reaction and 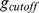 is a cutoff value. The choice of RMFs and two parameters, 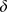 and 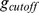, is condition-specific. In other words, depending on environmental conditions and organism characteristics, RMF can be biomass production, ATP production, or some other metabolic functionality; the appropriate values of 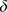 and 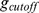 may also vary.

**Figure 1:**
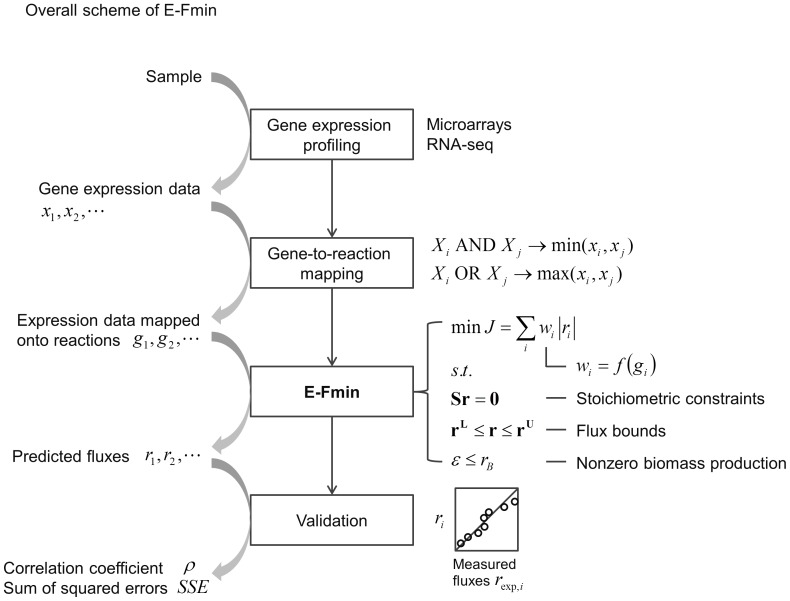
Schematic description of the E-Fmin framework. The algorithm was implemented through the following procedures. The first step was to obtain absolute gene expression profiles in a given condition from microarray, RNA-seq, or other high-throughput methods. Second, gene expression profiles were mapped onto individual reactions using gene-reaction associations. Third, the mapped expression data were integrated with the network model, and the optimization problem is solved to predict the flux distribution. Finally, model predictions were validated by comparison with experimentally measured flux data. The performance of model prediction can be gauged using standard measures, such as correlation coefficients (denoted by ρ) and sum of squared error (SSE).

To develop a tool that enables quantitative flux predictions, we made modifications to GIMME. First, we replaced *Eq. 4* with the constraint 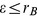
(5)where *r_B_* is the specific rate of biomass production and 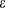 is an arbitrarily small value. *Eq. 5* allows the specific rate of biomass production to vary from a negligible value to its theoretical maximal value, and thus should be valid even under conditions of significantly suppressed growth. In this regard, we view *Eq. 5* as a context-independent constraint that can be applied across a wide range of conditions. It should be noted that 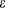 is not a parameter to be adjusted, as the final normalized results are invariant for any particular choice. Second, we set 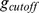 as the maximum value of gene expression data (i.e., unity value after normalization). Then, *w_i_* is represented as 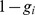, meaning that E-Fmin suppresses *all* reactions (except those fully expressed) in inverse proportion to their associated gene expression levels. For more details on the E-Fmin algorithm, see materials and methods. Figure 1 shows the overall procedure of implementing and assessing the performance of E-Fmin.

### Illustrative Example

**Figure 2:**
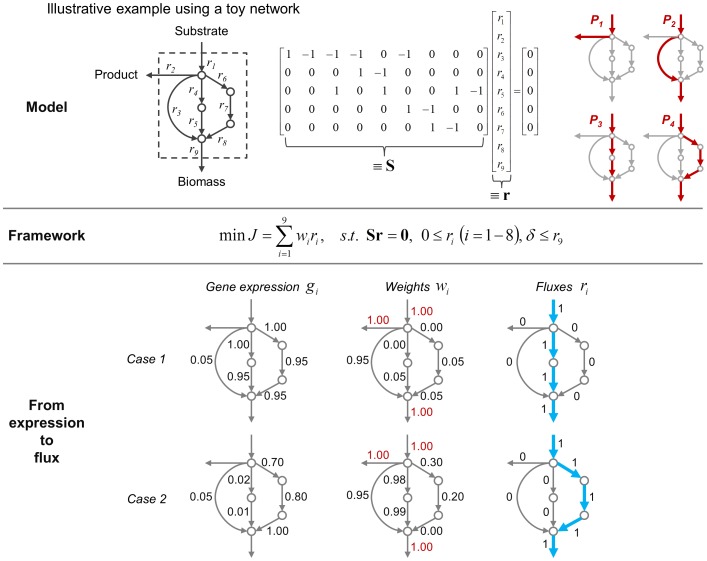
Toy example illustrating an implementation of the E-Fmin algorithm. The network model includes nine reactions (*r*_1_ to *r*_9_) but only five available stoichiometric constraints among the five intracellular metabolites under the steady-state assumption. E-Fmin determines the full flux vector for this undetermined system by solving a linear programming problem such that a weighted sum of flux magnitudes is minimized while biomass production (i.e., *r*_9_ in this example) carries nonzero flux. Given two sets of transcriptomic data, E-Fmin generates different flux distributions (denoted by thick arrows). The weight to the *i* ^th^ reaction (*w_i_*) is formulated as a decreasing function of the associated gene expression level (*g_i_*), i.e., *w_i_* = 1 – *g_i_*. The weights highlighted in red represent the reactions for which no associated gene expression data are available.

Figure 2 shows the application of E-Fmin to a toy metabolic network. The example network contains four major pathway options (*P*_1_ to *P*_4_) for converting substrate into product and biomass. Path *P*_1_ leads to product formation through reactions *r*_1_ and *r*_2_, whereas paths *P*_2_, *P*_3_, and *P*_4_ lead to biomass formation through reactions (*r*_1_, *r*_3_, and *r*_9_), (*r*_1_, *r*_4_, *r*_5_, and *r*_9_), and (*r*_1_, *r*_6_, *r*_7_, *r*_8_, and *r*_9_), respectively. This metabolic network is underdetermined, as it has nine unknown fluxes, *r*_1_ to *r*_9_, but only five available equations given by the stoichiometric matrix **S** in Figure 2. Thus, the determination of a particular flux distribution among infinite solutions requires either additional experimental flux measurements or application of a computational optimization method such as E-Fmin.

In the absence of gene expression data, E-Fmin treats all weights as equal, i.e., *w_i_* = 1 (*i* = 1,2, …, 9). E-Fmin then selects *P*_2_, the shortest among the biomass-producing pathways, as the solution. Note that *P*_1_, while shorter than *P*_2_, is not the solution of E-Fmin due to the constraint that forces biomass production to carry flux.

Incorporation of gene transcript expression levels into E-Fmin allows us to use biological and condition-specific information to estimate the flux distribution in the system. Figure 2 shows two different exemplar gene expression patterns (*cases 1* and *2*). The weights are given as a function of expression level, 1 – *g_i_*, where *g_i_* is the normalized gene expression level for the *i* ^th^ reaction, ranging from 0 to 1. We set the weights of the reactions for which no gene expression levels are available to 1. In *case 1*, E-Fmin discards *P*_2_ as the associated genes are not highly expressed. For paths *P*_3_ and *P*_4_, which are similar in their gene expression levels, E-Fmin selects the former because of the smaller sum of flux magnitudes. *Case 2* shows that even the longest pathway, *P*_4_, can be selected as the solution of E-Fmin if the associated relative gene expression levels are sufficiently high.

E-Fmin, like other LP-based algorithms, yields only a single pathway among alternative solutions, despite the likelihood that multiple routes can simultaneously be activated at a certain ratio according to their associated gene expression levels. For example, in *case 1*, it is probable that both *P*_3_ and *P*_4_ could carry nonzero throughput fluxes. To account for these situations, we would implement flux variability analysis (FVA) after the E-Fmin analysis to account for the range of flux variation through each reaction.

### Prediction of Exometabolomic Fluxes of *S. cerevisiae*

We applied our algorithm to the aerobic growth of *S. cerevisiae*. We used experimental data collected by Lee et al., which included RNA-seq transcriptomic data and exchange flux measurements of *S. cerevisiae* aerobically growing in chemostat cultures. This study provides data under two different growth conditions, i.e., glucose uptake fluxes of 16.5 and 11.0 mmol/(gDW⋅h), which correspond to 75% and 85% of the maximal attainable biomass levels, respectively. We used a genome-scale metabolic network of *S. cerevisiae*, Yeast 5 [28]. While earlier metabolic network reconstructions of yeast have been reported separately by different groups, Yeast 5 is a community-driven network reconstruction based on standard names and methods and is updated periodically.

**Figure 3:**
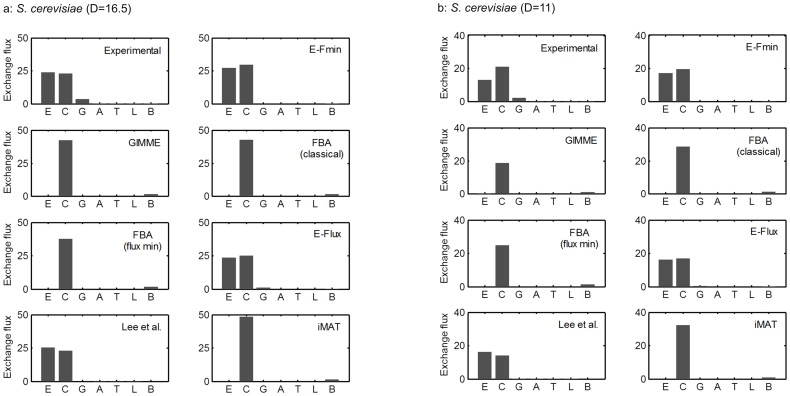
Exometabolome data of *Saccharomyces cerevisiae* and model predictions. Bars represent experimental and predicted values of exchange fluxes when the glucose uptake was **A**) 16.5 and **B**) 11.0 mmol/(gDW⋅h). Capital letters on the *x*-axis denote the production rates of extracellular metabolites, i.e., E: ethanol production, C: CO_2_ production, G: glycerol production, A: acetate production, T: trehalose production, L: lactose production, and B: biomass production. GIMME, Gene Inactivity Moderated by Metabolism and Expression; FBA, flux balance analysis; iMAT, integrative metabolic analysis tool.

Figures 3A and 3B show experimentally measured exchange fluxes and model predictions at glucose uptake fluxes of 16.5 and 11.0 mmol/(gDW⋅h), respectively. For comparison, we also show the predictions from other methods, including GIMME, FBA, E-Flux, Lee et al.'s algorithm [19], and iMAT, along with those of E-Fmin. We set the maximization of biomass production as the objective of FBA and implemented it in two different forms: without flux minimization (or classical) and with flux minimization. For GIMME and iMAT, we constrained the production of biomass to be above a certain threshold (90% of the maximal growth rate as predicted by FBA), which prevents the predicted growth rate from becoming zero. See materials and methods for details on their implementation.

The experimental data showed that ethanol and CO_2_ are the predominant products, whereas the production of glycerol, acetate, trehalose, lactose, and biomass is insignificant. This trend is well captured by E-Fmin, E-Flux, and Lee et al., but not by GIMME, FBA, and iMAT. Table 1 and Table S1 show correlation coefficients (denoted by ρ in this report) and sum of squared error (SSE) values, respectively, obtained by comparison between model predictions and experimental data. E-Fmin, E-Flux, and Lee et al. show higher ρ values (Table 1) and lower SSE values (Table S1) in comparison to other methods. As detailed in the Materials and Methods, we obtained a P value of 0.04 and rejected the null hypothesis that the ranking of average correlation coefficients in Table 1 could occur by chance alone. The ability to accurately predict the production of metabolic products (such as ethanol) is important, as they are often the target products of interest for biotechnological applications. The poor prediction of FBA implies that maximization of biomass production may not be a valid assumption for these specific experimental conditions for the yeast growth. Whereas the performance of GIMME (and iMAT) may be improved by adjusting their parameters, their appropriate values cannot be determined *a priori*. We provided the flux data and model predictions in Table S2.

**Table 1:** Correlation coefficients (ρ) and P values of E-Fmin and other methods in predicting exchange fluxes and growth rate in *Saccharomyces cerevisiae* with different uptake fluxes for glucose.

Uptake Rate, mmol/(gDW⋅h)	E-Fmin	GIMME	FBA (classical)	FBA (flux min)	E-Flux	Lee et al.	iMAT
16.5	0.99 (5×10^−6^)	0.64 (8×10^−2^)	0.64 (9×10^−2^)	0.64 (8×10^−2^)	0.99 (3×10^−7^)	0.99 (7×10^−7^)	0.63 (9×10^−2^)
11.0	0.98 (3×10^−5^)	0.83 (1×10^−2^)	0.83 (1×10^−2^)	0.83 (1×10^−2^)	0.97 (1×10^−4^)	0.93 (7×10^−4^)	0.83 (1×10^−2^)
**Average**	**0.98**	**0.74**	**0.74**	**0.74**	**0.98**	**0.96**	**0.73**

FBA, flux balance analysis; GIMME, Gene Inactivity Moderated by Metabolism and Expression; iMAT, integrative metabolic analysis tool.

### Prediction of Intracellular Flux Distribution in *E. coli*

To test our algorithm across organisms, we additionally applied it to the aerobic growth of *E. coli* in chemostat cultures. We obtained microarray gene expression profiles (from central carbon metabolism) and ^13^C-MFA-based flux data of *E. coli* K-12 strains from Ishii et al. [29]. In their work, Ishii et al. experimentally investigated the response of *E. coli* to environmental and genetic perturbations and provided multiple high-throughput omics data for both wild-type and mutant strains. To study the effect of environmental perturbations, they cultured wild-type cells at varied dilution rates, while the effect of genetic perturbations was examined by disrupting 24 single genes contained in glycolysis and in the pentose phosphate pathway. As a result, they observed that gene disruptions lead to only subtle changes in mRNA levels, suggesting that *E. coli* is able to adequately compensate for the loss of a single gene or enzyme in the central metabolism by using excess or complementary capacity of other available enzymes. Conversely, they reported that wild-type *E. coli* cells appreciably change mRNA levels in response to the variation of the dilution rates but exhibit low-variations (robustness) in metabolite concentration levels. Figure S1 shows that the experimentally measured flux distributions are also robust against such environmental perturbations. Therefore, this system poses a challenging problem in integrating varying gene expression profiles for different dilution rates yet predicting an unchanging flux distribution.

For computational predictions, we incorporated the gene expression data of Ishii et al. and an *E. coli* network model into the E-Fmin framework as detailed in materials and methods. Multiple genome scale metabolic reconstructions of *E. coli* have been built in the last decade [30], and, in this study, we used a recent, well-curated genome-scale network of *E. coli* K-12 strain, iAF1260 [31]. As an updated version of the previous reconstruction iJR904 [32], iAF1260 comprehensively accounts for 1,260 open reading frames, which correspond to ∼30% of *E. coli*'s genome [33].

**Figure 4:**
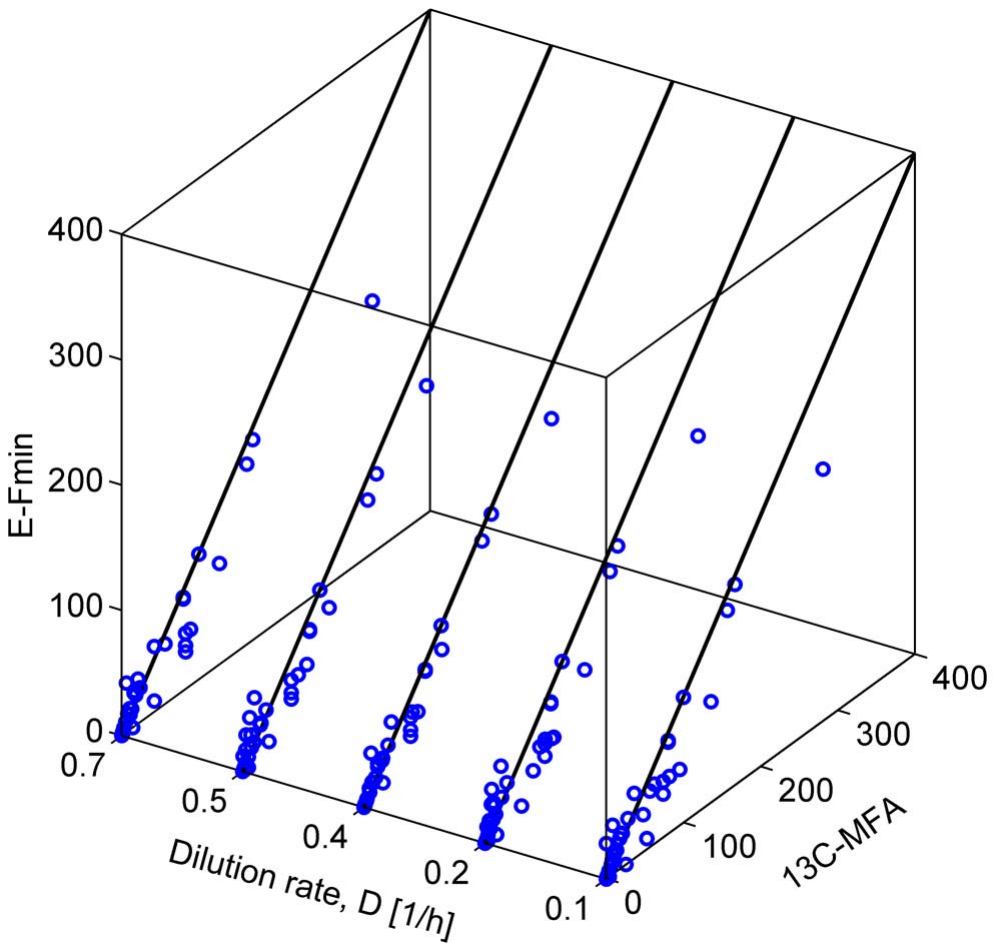
E-Fmin predictions of intracellular metabolic fluxes in wild-type *Escherichia coli*. Shown is a comparison of the metabolic fluxes at varied dilution rates (D; *x*-axis) as measured using ^13^C-metabolic flux analysis (^13^C-MFA; *y*-axis) and predicted by E-Fmin (*z*-axis). In all cases, flux comparisons were made using their relative values normalized with the glucose uptake flux of 100 mmol/(gDW⋅h).

Figure 4 shows intracellular metabolic fluxes of wild-type *E. coli* strains obtained from E-Fmin and ^13^C-MFA at respective dilution rates. Predicted fluxes show good matches with ^13^C-MFA data in all cases. Table 2 shows ρ with an average value of 0.91 along with *P* values. Low *P* values indicate that the correlations obtained from E-Fmin are statistically significant. Table S1 shows relatively low values of SSE with an average value of 57.8. We observed that E-Fmin underestimated the CO_2_ production rate in all cases.

**Table 2:** Correlation coefficients (ρ) and P values of E-Fmin and other methods in predicting intracellular flux distributions in wild-type *Escherichia coli* at varied dilution rates.

Dilution Rate, 1/h	E-Fmin	GIMME	FBA (classical)	FBA (flux min)	E-Flux	Lee et al.	iMAT	iMAT (outliers removed)
0.1	0.91 (1×10^−17^)	0.86 (1×10^−13^)	<0.01 (1×10^0^)	0.94 (4×10^−20^)	0.46 (2×10^−3^)	0.35 (2×10^−2^)	<0.01 (1×10^0^)	0.88 (2×10^−14^)
0.2	0.88 (8×10^−15^)	0.86 (3×10^−13^)	0.04 (8×10^−1^)	0.93 (6×10^−20^)	0.40 (8×10^−3^)	0.40 (9×10^−3^)	0.04 (8×10^−1^)	0.85 (6×10^−13^)
0.4	0.91 (9×10^−17^)	0.91 (5×10^−16^)	0.05 (8×10^−1^)	0.97 (4×10^−26^)	0.66 (3×10^−6^)	0.43 (5×10^−3^)	0.05 (8×10^−1^)	0.91 (9×10^−16^)
0.5	0.94 (4×10^−20^)	0.86 (8×10^−13^)	0.05 (7×10^−1^)	0.95 (6×10^−21^)	0.77 (6×10^−9^)	0.43 (6×10^−3^)	0.05 (7×10^−1^)	0.85 (6×10^−12^)
0.7	0.92 (1×10^−17^)	0.83 (3×10^−11^)	0.08 (6×10^−1^)	0.93 (1×10^−18^)	0.84 (1×10^−11^)	0.21 (2×10^−1^)	0.08 (6×10^−1^)	0.90 (3×10^−15^)
**Average**	**0.91**	**0.86**	**0.04**	**0.94**	**0.62**	**0.36**	**0.04**	**0.88**

FBA, flux balance analysis; GIMME, Gene Inactivity Moderated by Metabolism and Expression; iMAT, integrative metabolic analysis tool.

Table 2 also shows the performance results for the other algorithms investigated. Similar to the statistical analysis of Table 1, we obtained a P value of <10^−10^ and rejected the null hypothesis that the ranking of average correlation coefficients in Table 2 could arise by chance alone. The classical FBA (without flux minimization) performed poorly because some of the fluxes attained their upper (or lower) bound values, a problem associated with the existence of multiple LP solutions. These outliers were removed by applying the flux minimization as a secondary objective. Consequently, with the flux minimization, FBA provides reliable estimates, implying that the hypothesis of maximal biomass production may be valid for *E. coli* growing under these specific conditions. Interestingly, despite using the same objective function, the prediction of E-Flux was not comparable to that of FBA. It seems that the E-Flux predictions in this system are more affected by the imposed flux bounds than the choice of objective function. Algorithms based on the direct association with expression levels, such as in Lee et al., showed weak predictive powers, which may be ascribed in part to the use of gene expression data covering only the central carbon metabolism. Similarly to the classical FBA, the flux vector predicted by iMAT also contained outlier fluxes that reached their bounds. After removing them, iMAT showed an improved predictive capability. We provided the intracellular flux data and model predictions at the dilution rate of 0.1 1/h in Table S3.

Table 3 shows similar results for the 24 single-gene knockout mutants; i.e., E-Fmin, GIMME, FBA (with flux minimization), and iMAT calculations were associated with correlation coefficient averages of 0.87, 0.84, 0.92, and 0.86, respectively, whereas the other algorithms exhibited relatively lower average correlation coefficients ranging from 0.05 to 0.53. We obtained a P value of <10^−10^ and rejected the null hypothesis that the ranking of average correlation coefficients in Table 3 could arise by chance alone. Performance comparison using SSE values in Table S1 showed the same trend. The satisfactory performance results of GIMME and iMAT were due to the imposed biomass production constraint. The correlation coefficients (and SSE) values became lower (and higher) for both methods when the threshold was adjusted to a lower value (results not shown).

**Table 3:** Correlation coefficients ρ (and *P* values) of E-Fmin and other methods in predicting metabolic fluxes in mutant *Escherichia coli* strains with single gene knockouts.

Knockout Gene	E-Fmin	GIMME	FBA (classical)	FBA (flux min)	E-Flux	Lee et al.	iMAT (outliers removed)
galM	0.90 (2×10^−16^)	0.85 (5×10^−13^)	0.01 (9×10^−1^)	0.93 (5×10^−19^)	0.40 (8×10^−3^)	0.36 (2×10^−2^)	0.87 (5×10^−14^)
glk	0.84 (2×10^−12^)	0.82 (2×10^−11^)	0.08 (6×10^−1^)	0.91 (2×10^−17^)	0.52 (3×10^−4^)	0.37 (2×10^−2^)	0.83 (8×10^−12^)
pgm	0.87 (7×10^−14^)	0.82 (2×10^−11^)	0.06 (7×10^−1^)	0.93 (8×10^−19^)	0.72 (6×10^−8^)	0.38 (1×10^−2^)	0.85 (7×10^−13^)
pgi	0.93 (6×10^−19^)	0.84 (3×10^−12^)	−0.03 (9×10^−1^)	0.90 (4×10^−16^)	0.56 (9×10^−5^)	0.23 (2×10^−1^)	0.93 (8×10^−19^)
pfkA	0.87 (4×10^−14^)	0.86 (1×10^−13^)	0.05 (7×10^−1^)	0.94 (2×10^−20^)	0.70 (2×10^−7^)	0.44 (3×10^−3^)	0.84 (2×10^−12^)
pfkB	0.93 (2×10^−19^)	0.88 (5×10^−15^)	−0.01 (9×10^−1^)	0.95 (1×10^−21^)	0.58 (4×10^−5^)	0.35 (2×10^−2^)	0.91 (2×10^−16^)
fbp	0.88 (6×10^−15^)	0.85 (4×10^−13^)	−0.01 (9×10^−1^)	0.92 (7×10^−18^)	0.47 (2×10^−3^)	0.42 (5×10^−3^)	0.82 (3×10^−11^)
fbaB	0.89 (8×10^−16^)	0.87 (2×10^−14^)	0.03 (8×10^−1^)	0.95 (2×10^−21^)	0.53 (3×10^−4^)	0.41 (7×10^−3^)	0.87 (8×10^−14^)
gapC	0.91 (4×10^−17^)	0.86 (2×10^−13^)	−0.01 (1×10^0^)	0.93 (5×10^−19^)	0.52 (4×10^−4^)	0.36 (2×10^−2^)	0.87 (5×10^−14^)
gpmA	0.87 (3×10^−14^)	0.84 (1×10^−12^)	0.05 (7×10^−1^)	0.94 (7×10^−21^)	0.53 (2×10^−4^)	0.43 (4×10^−3^)	0.85 (1×10^−12^)
gpmB	0.83 (5×10^−12^)	0.80 (1×10^−10^)	0.09 (6×10^−1^)	0.90 (2×10^−16^)	0.55 (1×10^−4^)	0.36 (2×10^−2^)	0.83 (1×10^−11^)
pykA	0.89 (1×10^−15^)	0.86 (1×10^−13^)	0.03 (9×10^−1^)	0.94 (4×10^−20^)	0.56 (9×10^−5^)	0.42 (6×10^−3^)	0.86 (3×10^−13^)
pykF	0.94 (4×10^−21^)	0.90 (5×10^−16^)	−0.03 (8×10^−1^)	0.95 (1×10^−21^)	0.59 (3×10^−5^)	0.34 (3×10^−2^)	0.91 (3×10^−17^)
ppsA	0.87 (2×10^−14^)	0.86 (3×10^−13^)	0.08 (6×10^−1^)	0.94 (2×10^−21^)	0.35 (2×10^−2^)	0.38 (1×10^−2^)	0.89 (4×10^−15^)
zwf	0.85 (7×10^−13^)	0.90 (4×10^−16^)	0.04 (8×10^−1^)	0.90 (1×10^−15^)	0.59 (3×10^−5^)	0.38 (1×10^−2^)	0.82 (4×10^−11^)
pgl	0.88 (5×10^−15^)	0.85 (3×10^−13^)	0.05 (8×10^−1^)	0.93 (7×10^−20^)	0.72 (4×10^−8^)	0.39 (9×10^−3^)	0.85 (3×10^−13^)
gnd	0.84 (2×10^−12^)	0.89 (9×10^−16^)	0.07 (6×10^−1^)	0.90 (5×10^−16^)	0.46 (2×10^−3^)	0.36 (2×10^−2^)	0.83 (8×10^−12^)
rpe	0.87 (2×10^−14^)	0.79 (4×10^−10^)	0.07 (6×10^−1^)	0.89 (4×10^−15^)	0.64 (4×10^−6^)	0.31 (5×10^−2^)	0.82 (3×10^−11^)
rpiA	0.86 (1×10^−13^)	0.80 (1×10^−10^)	0.06 (7×10^−1^)	0.90 (2×10^−16^)	0.73 (3×10^−8^)	0.28 (7×10^−2^)	0.84 (2×10^−12^)
rpiB	0.85 (5×10^−13^)	0.79 (3×10^−10^)	0.09 (6×10^−1^)	0.90 (3×10^−16^)	0.44 (3×10^−3^)	0.22 (2×10^−1^)	0.88 (8×10^−15^)
tktA	0.79 (2×10^−10^)	0.73 (1×10^−8^)	0.14 (4×10^−1^)	0.86 (8×10^−14^)	0.30 (5×10^−2^)	0.25 (1×10^−1^)	0.82 (2×10^−11^)
tktB	0.84 (1×10^−12^)	0.83 (4×10^−12^)	0.09 (6×10^−1^)	0.93 (8×10^−19^)	0.49 (9×10^−4^)	0.41 (7×10^−3^)	0.84 (3×10^−12^)
talA	0.86 (3×10^−13^)	0.85 (5×10^−13^)	0.08 (6×10^−1^)	0.93 (6×10^−20^)	0.53 (2×10^−4^)	0.43 (4×10^−3^)	0.84 (2×10^−12^)
talB	0.85 (8×10^−13^)	0.84 (3×10^−12^)	0.08 (6×10^−1^)	0.93 (6×10^−19^)	0.36 (2×10^−2^)	0.40 (9×10^−3^)	0.84 (3×10^−12^)
**Average**	**0.87**	**0.84**	**0.05**	**0.92**	**0.53**	**0.36**	**0.86**

The gene names in the first column of the table denote mutants with the corresponding gene disrupted.

FBA, flux balance analysis; GIMME, Gene Inactivity Moderated by Metabolism and Expression; iMAT, integrative metabolic analysis tool.

### Prediction of Biomass Yield

As demonstrated in the case studies considered above, E-Fmin has strong predictive capabilities for the metabolism of both *S. cerevisiae* and *E. coli* despite the different metabolic characteristics of these organisms. For *E. coli*, the assumption of maximal production of biomass underlies the successful application of FBA in many studies of this organism [34], [35]. Experimentally measured biomass yields of *E. coli* at balanced growth conditions are close to the theoretical maximum predicted by FBA [36]. Conversely, the metabolism of more advanced organisms, including yeast, may not be adequately accounted for by the same hypothesis [20]. Accordingly, the biomass yield of yeast is much lower than its theoretical maximal value.

The E-Fmin algorithm was designed to account for all of these features. For *E. coli*, E-Fmin predicted biomass yield of 0.095 gDW/(mmol-glucose) for both wild-type and mutant *E. coli* strains, which is close to the FBA prediction of 0.096 gDW/(mmol-glucose), but without requiring biomass production to be maximized. In the case of yeast, E-Fmin predicted a biomass yield of 0.028 gDW/(mmol-glucose) for both uptake fluxes of 16.5 and 11.5 mmol/(gDW⋅h), which correspond to ∼25% of the theoretical maximum 0.104 gDW/(mmol-glucose). While Crabtree-negative yeast strains, such as *Pichia stipitis*, show high biomass yield close to the FBA prediction, the biomass yield of Crabtree-positive yeast *S. cerevisiae* is known to be much lower due to appreciable production of fermentation products, particularly ethanol [37]. The experimentally obtained biomass yields by Lee et al. were 0.020 gDW/(mmol-glucose) for both systems, confirming the accuracy of the E-Fmin predictions. The low biomass yield of *S. cerevisiae* growing in aerobic cultures has also been observed in other experiments. For instance, Papini et al. [37] experimentally measured the biomass yield of wild-type *S. cerevisiae* growing on glucose as 0.031 gDW/(mmol-glucose), which is close to our prediction. Conversely, the algorithm by Lee et al. predicted zero biomass yields for both *E. coli* and *S. cerevisiae*. The prediction of iMAT also led to zero biomass yields without additional constraint for biomass production to be larger than a certain level (e.g., 90% in our simulations) of the theoretical maximal.

### Features of the E-Fmin Algorithm

The main distinguishing features of the E-Fmin algorithm provide multiple advantages when analyzing cellular metabolism based on gene expression data. First, E-Fmin can use a generic network model to predict context-specific flux distribution. This is not the case for other methods, such as EXAMO, where the reconstruction of a condition-specific subnetwork is a prerequisite. Second, E-Fmin uses absolute gene expression data that can be collected directly from a single condition without defining or determining a standard reference conditions. This is advantageous over many of the currently available methods that are based on relative expression data [14]–[16], which typically require expanded sets of data including flux distribution at a reference condition. Conversely, if the reference flux distribution is not known, E-Fmin can be used to provide the reference flux distribution. Third, E-Fmin contains no parameters to adjust. Note that any arbitrary positive value can be used for ε in *Eq. 5*. The flux distribution obtained as the solution of E-Fmin becomes the same after normalization regardless of the value of ε. The normalized flux vector (obtained from E-Fmin) can be scaled to ‘actual’ values using experimentally measured fluxes, whenever available. Finally, the formulated LP problem leads to fast simulations. This is an important consideration when performing extensive FVA or analyzing systems for a large number of conditions.

In addition, due to the principle of flux minimization, fluxes predicted by E-Fmin contain no thermodynamically infeasible cyclic paths. The flux minimization also shrinks the space of alternative optimal solutions, leading to very narrow flux variation through individual reactions. We observed no appreciable changes in E-Fmin predictions from the implementation of FVA.

In all simulation studies including E-Fmin, we assumed that the cellular composition was known. For cases in which cells grow in conditions that significantly differ from standard cultures, additional measurement of biomass composition is likely to improve model predictions.

### Conclusions

The work reported in this article addresses the issue of how one can effectively use gene expression data to study a phenotypic response of metabolism under various conditions by estimating flux distribution. Although a direct link between transcriptomic and fluxomic data is generally weak due to posttranscriptional modifications, the framework we developed was able to extract maximal information of metabolic fluxes by integrating mRNA data with a metabolic network model based on the principle of flux minimization. Importantly, E-Fmin does not require *a priori* knowledge of context-specific metabolic functionalities. This feature allows E-Fmin to be applicable across different conditions/organisms without modifying any components of the framework. We validated this capability through studies of *S. cerevisiae* and *E. coli* strains exhibiting distinct metabolic characteristics. In comparing the overall ability of the different methods to achieve a ranking above average, i.e., a meta-analysis of all rankings from the separate Tables 1–3, we found that both E-Fmin and FBA (flux min) were statistically significantly ranked above all other methods (P values 0.03 and 0.04, respectively). Although similar in nature and performance, this analysis misses the fact that the latter method was unable to give satisfactory results for the *Saccharomyces cerevisiae* data. Thus, compared with other methods, E-Fmin provided more consistently reliable predictions for both organisms. While these studies used gene expression measurements, E-Fmin could incorporate protein expression data as an alternative input to predict flux distribution.

As a basic constraint, E-Fmin forced nonzero flux through the biomass-producing reaction. This constraint was sufficient for E-Fmin to be able to correctly predict biomass production, high in *E. coli* and low in *S. cerevisiae*, based on absolute gene expression data. Nevertheless, the inclusion of an expanded set of key reactions may be required to obtain physiologically reliable prediction for a specific condition. A systematic basis for the identification of additional condition-specific constraints is therefore a topic of great general interest. In our future work, we will expand the applications of the E-Fmin framework to examine a broader range of physiological conditions.

## Materials and Methods

### Metabolic Network Models and Experimental Data

We used genome-scale metabolic network models taken from the recent *E. coli* and *S. cerevisiae* reconstructions iAF1260 [31] and Yeast 5 [28], respectively. We downloaded the Systems Biology Markup Language (SBML) model of iAF1260 from the Biochemical Genetic and Genomic knowledgebase [38], which contains 2,382 reactions, 1,668 metabolites, and 1,261 genes. We obtained the SBML model of Yeast 5 (version 5.21) from Lee et al. [19], which contains 2,061 reactions, 1,605 metabolites, and 893 genes. To evaluate the prediction of our algorithm using a minimum number of auxiliary metabolic information, we removed any condition/organism-specific constraints, such as ATP requirements for cellular maintenance and oxygen/carbon uptake rates. We allowed the magnitudes of all fluxes to vary without bounds.

We obtained preprocessed gene expression profiles and ^13^C-MFA data of *E. coli* from Ishii et al. [29]. They provided wild-type strain data at several different dilution rates (i.e., 0.1, 0.2, 0.4, 0.5, and 0.7 1/h) and mutant strain data for 24 single-gene disruptions at a fixed dilution rate of 0.2 1/h. We similarly obtained data for *S. cerevisiae* from Lee et al. [19], which included preprocessed gene expression data, growth rate, and exchange flux measurements, including the uptake rate of glucose and the production rates of ethanol, CO_2_, glycerol, acetate, trehalose, and lactose. In both experiments, data were collected from glucose-limited chemostat cultures.

### Gene-To-Reaction Mapping and Further Processing

The initial step in predicting fluxes using E-Fmin is to map gene expression data onto reactions based on gene-protein-reaction associations, which are provided with the iAF1260 and Yeast 5 network models. This mapping is straightforward if a single gene product catalyzes a reaction, i.e., the association of a gene via mRNA and enzyme to a single reaction is unambiguous. In general, a reaction is associated with multiple gene products, and their relations are described using Boolean operators such as “AND” and “OR.” The AND operation represents the involvement of multiple gene products in catalyzing a reaction, whereas the OR operation signifies that only one of the gene products is involved in the reactions. We implemented these operations by taking the minimal and maximal value of the associated gene expression data [39], as follows: 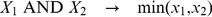
(6)

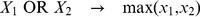
(7)where uppercase and lowercase letters indicate gene names and their expression levels. After this mapping, we divided the expression data by their maximum value to normalize their ranges from 0 to 1. We used *g_i_* to denote the resulting normalized gene expression values for the *i* ^th^ reaction.

We designed *w_i_* in *Eq. 1* as a linearly decreasing function of 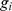 as follows: 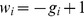
(8)


*Eq. 8* ensures that fluxes with lower (higher) gene expression levels are more (less) suppressed.

### Implementation

We recast the absolute-sum minimization problem presented in *Eqs. 1*–*3* and *5* into a linear form, as follows: 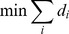
(9)subject to
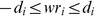
(10)in addition to the constraints given in *Eqs. 2*, 3, and *5*. We used 0.01 for ε in *Eq. 5*.

We solved the above problem using the CPLEX (ILOG, Mountain View, CA) LP solver. We ran all simulations on a desktop PC with an Intel (Santa Clara, CA) Pentium i3 CPU and 4-GM RAM. We provide the MATLAB scripts used for generating the results in Data Set S1. We used our own code for the implementation of E-Fmin and GIMME, the COBRA package for FBA and E-flux, and Lee et al.'s scripts for their algorithm and iMAT. For the implementation of GIMME, we constrained the biomass production to be greater than or equal to 90% of the theoretical maximum and set 0.05 as the cutoff value (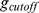). Data Sets S2 and S3 provide the network models and data files used for the simulations of *S. cerevisiae* and *E. coli* metabolism, respectively.

### Statistical Analysis of Correlation Tables

Given a table of correlation coefficients of predicted intracellular flux distributions for various conditions and methods, we wanted to test whether the observed rankings could occur by chance. We first normalized the correlation coefficients to scores ranging from 0 to 1 by rank-ordering all of them from small to large and, for each observation, scoring its rank as a percentile. Then, we used the F-test in a one-way analysis of variance [40] to ascertain significance. In short, we computed the F-ratio of between-method variability (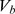) to within-method variability (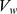):
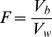
(11)


The variability values were calculated as
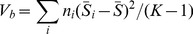
(12)and
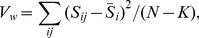
(13)where 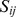 is the score of method *i* in condition *j*, 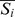
denotes the average of the scores of method *i* in all conditions, 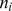 is the number of conditions for method *i*, 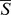
denotes the overall average, 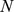
is the number of observations, and 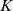 is the number of methods. Using the F-ratio, between-method degree 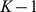, and within-method degree 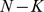, we determined the P value according to the F-distribution.

Finally, we used rank product analysis [41] to assess whether any method performed significantly above average among all tested methods. For each of the Tables 1–3, we sorted the methods according to their average scores from large to small and obtained their ranks. We computed the geometric mean of the ranks of each method of Tables 1–3 as its rank product *RP*:
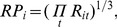
(14)where 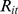 is the rank of method *i* in table *t*. We exhaustively permuted the three, sorted lists, and computed the P value as the fraction of all permutations that had smaller or equal rank products.

## Supporting Information

Figure S1 **Wild-type** ***Escherichia coli*** **data collected at different dilution rates (Ishii et al., Science, 2007): gene expression data (triangles;** ***A*** **), flux data (circles;** ***B*** **), principal component analysis (PCA) using gene expression data (triangles;** ***C*** **), and flux data (circles; D).** Gene expression data were normalized to range from 0 to 1. Flux data were scaled so that the glucose uptake flux is 100 mmol/(gDW⋅h). For the PCA, both gene expression and flux data were normalized by their maximal value to range from 0 to 1. D denotes dilution rate [1/h].
(TIFF)

Table S1 **Sum of squared error (SSE) of E-Fmin and other methods in predicting intracellular flux distributions in** ***Saccharomyces cerevisiae*** **at different glucose uptake rates, wild-type** ***Escherichia coli*** **at varied dilution rates, and mutated** ***E. coli*** **with single gene knockouts.**
(DOCX)

Table S2 **Comparison of exchange fluxes of** ***Saccharomyces cerevisiae*** **between experimental data and computational predictions.**
(DOCX)

Table S3 **Comparison of intracellular fluxes of wild-type** ***Escherichia coli*** **at a dilution rate (D) of 0.1 (1/h) between experimental data and computational predictions.**
(DOCX)

Data Set S1 **Matlab scripts.** Provides numeric codes for running E-Fmin and other algorithms to predict flux distributions from given gene expression profiles. At the end of the simulation, the code calculates correlation coefficients (ρ) together with *P* values and coefficients of determination (*R* ^2^) as performance measures for comparison.
(ZIP)

Data Set S2 **Network model and data files used for simulations of** ***Saccharomyces cerevisiae*** **metabolism.** Provides a zipped file containing a genome-scale metabolic network model of *S. cerevisiae* (Yeast 5) and two sets of gene expression and flux data for two different uptake fluxes of glucose.
(ZIP)

Data Set S3 **Network model and data files used for simulations of** ***Escherichia coli*** **metabolism.** Provides a zipped file containing a genome-scale metabolic network model of *E. coli* (iAF1260), five sets of gene expression and flux data for the wild-type strain, and 24 sets of the same for the mutant strains.
(ZIP)

## References

[1] Matsushika A, Inoue H, Kodaki T, Sawayama S (2009) Ethanol production from xylose in engineered *Saccharomyces cerevisiae* strains: current state and perspectives. Appl Microbiol Biotechnol 84: 37–53. 19572128 10.1007/s00253-009-2101-x

[2] Kim HU, Sohn SB, Lee SY (2012) Metabolic network modeling and simulation for drug targeting and discovery. Biotechnol J 7: 330–342. 22125297 10.1002/biot.201100159

[3] Chiaradonna F, Moresco RM, Airoldi C, Gaglio D, Palorini R, et al (2012) From cancer metabolism to new biomarkers and drug targets. Biotechnol Adv 30: 30–51. 21802503 10.1016/j.biotechadv.2011.07.006

[4] Bordbar A, Feist AM, Usaite-Black R, Woodcock J, Palsson BO, et al (2011) A multi-tissue type genome-scale metabolic network for analysis of whole-body systems physiology. BMC Syst Biol 5: 180. 22041191 10.1186/1752-0509-5-180 PMC3219569

[5] Zamboni N, Fendt SM, Ruhl M, Sauer U (2009) ^13^C-based metabolic flux analysis. Nat Protoc 4: 878–892. 19478804 10.1038/nprot.2009.58

[6] Sauer U (2006) Metabolic networks in motion: ^13^C-based flux analysis. Mol Syst Biol 2: 62. 17102807 10.1038/msb4100109 PMC1682028

[7] Gianchandani EP, Chavali AK, Papin JA (2010) The application of flux balance analysis in systems biology. Wiley Interdiscip Rev Syst Biol Med 2: 372–382. 20836035 10.1002/wsbm.60

[8] Oberhardt MA, Palsson BO, Papin JA (2009) Applications of genome-scale metabolic reconstructions. Mol Syst Biol 5: 320. 19888215 10.1038/msb.2009.77 PMC2795471

[9] Jerby L, Ruppin E (2012) Predicting drug targets and biomarkers of cancer via genome-scale metabolic modeling. Clin Cancer Res 18: 5572–5584. 23071359 10.1158/1078-0432.CCR-12-1856

[10] Blazier AS, Papin JA (2012) Integration of expression data in genome-scale metabolic network reconstructions. Front Physiol 3: 299. 22934050 10.3389/fphys.2012.00299 PMC3429070

[11] Akesson M, Forster J, Nielsen J (2004) Integration of gene expression data into genome-scale metabolic models. Metab Eng 6: 285–293. 15491858 10.1016/j.ymben.2003.12.002

[12] Hyduke DR, Lewis NE, Palsson BO (2013) Analysis of omics data with genome-scale models of metabolism. Mol Biosyst 9: 167–174. 23247105 10.1039/c2mb25453k PMC3594511

[13] Covert MW, Knight EM, Reed JL, Herrgard MJ, Palsson BO (2004) Integrating high-throughput and computational data elucidates bacterial networks. Nature 429: 92–96. 15129285 10.1038/nature02456

[14] Shlomi T, Cabili MN, Herrgard MJ, Palsson BO, Ruppin E (2008) Network-based prediction of human tissue-specific metabolism. Nat Biotechnol 26: 1003–1010. 18711341 10.1038/nbt.1487

[15] Fang X, Wallqvist A, Reifman J (2012) Modeling phenotypic metabolic adaptations of *Mycobacterium tuberculosis* H37Rv under hypoxia. PLOS Comput Biol 8: e1002688. 23028286 10.1371/journal.pcbi.1002688 PMC3441462

[16] Vital-Lopez FG, Wallqvist A, Reifman J (2013) Bridging the gap between gene expression and metabolic phenotype via kinetic models. BMC Syst Biol 7: 63. 23875723 10.1186/1752-0509-7-63 PMC3733621

[17] Colijn C, Brandes A, Zucker J, Lun DS, Weiner B, et al (2009) Interpreting expression data with metabolic flux models: predicting *Mycobacterium tuberculosis* mycolic acid production. PLOS Comput Biol 5: e1000489. 19714220 10.1371/journal.pcbi.1000489 PMC2726785

[18] Zur H, Ruppin E, Shlomi T (2010) iMAT: an integrative metabolic analysis tool. Bioinformatics 26: 3140–3142. 21081510 10.1093/bioinformatics/btq602

[19] Lee D, Smallbone K, Dunn WB, Murabito E, Winder CL, et al (2012) Improving metabolic flux predictions using absolute gene expression data. BMC Syst Biol 6: 73. 22713172 10.1186/1752-0509-6-73 PMC3477026

[20] Schuster S, Pfeiffer T, Fell DA (2008) Is maximization of molar yield in metabolic networks favoured by evolution? J Theor Biol 252: 497–504. 18249414 10.1016/j.jtbi.2007.12.008

[21] Ramkrishna D, Song HS (2012) Dynamic models of metabolism: review of the cybernetic approach. AIChE J 58: 986–997.

[22] Holzhutter HG (2004) The principle of flux minimization and its application to estimate stationary fluxes in metabolic networks. Eur J Biochem 271: 2905–2922. 15233787 10.1111/j.1432-1033.2004.04213.x

[23] Schuetz R, Kuepfer L, Sauer U (2007) Systematic evaluation of objective functions for predicting intracellular fluxes in *Escherichia coli* . Mol Syst Biol 3: 119. 17625511 10.1038/msb4100162 PMC1949037

[24] Schuetz R, Zamboni N, Zampieri M, Heinemann M, Sauer U (2012) Multidimensional optimality of microbial metabolism. Science 336: 601–604. 22556256 10.1126/science.1216882

[25] Ramkrishna D, Kompala DS, Tsao GT (1987) Are microbes optimal strategists? Biotechnol Prog 3: 121–126.

[26] Rossell S, Huynen MA, Notebaart RA (2013) Inferring metabolic states in uncharacterized environments using gene-expression measurements. PLOS Comput Biol 9: e1002988. 23555222 10.1371/journal.pcbi.1002988 PMC3605102

[27] Becker SA, Palsson BO (2008) Context-specific metabolic networks are consistent with experiments. PLOS Comput Biol 4: e1000082. 18483554 10.1371/journal.pcbi.1000082 PMC2366062

[28] Heavner BD, Smallbone K, Barker B, Mendes P, Walker LP (2012) Yeast 5 – an expanded reconstruction of the *Saccharomyces cerevisiae* metabolic network. BMC Syst Biol 6: 55. 22663945 10.1186/1752-0509-6-55 PMC3413506

[29] Ishii N, Nakahigashi K, Baba T, Robert M, Soga T, et al (2007) Multiple high-throughput analyses monitor the response of *E. coli* to perturbations. Science 316: 593–597. 17379776 10.1126/science.1132067

[30] McCloskey D, Palsson BO, Feist AM (2013) Basic and applied uses of genome-scale metabolic network reconstructions of *Escherichia coli* . Mol Syst Biol 9: 661. 23632383 10.1038/msb.2013.18 PMC3658273

[31] Feist AM, Henry CS, Reed JL, Krummenacker M, Joyce AR, et al (2007) A genome-scale metabolic reconstruction for *Escherichia coli* K-12 MG1655 that accounts for 1260 ORFs and thermodynamic information. Mol Syst Biol 3: 121. 17593909 10.1038/msb4100155 PMC1911197

[32] Reed JL, Vo TD, Schilling CH, Palsson BO (2003) An expanded genome-scale model of *Escherichia coli* K-12 (iJR904 GSM/GPR). Genome Biol 4: R54. 12952533 10.1186/gb-2003-4-9-r54 PMC193654

[33] Thiele I, Fleming RM, Que R, Bordbar A, Diep D, et al (2012) Multiscale modeling of metabolism and macromolecular synthesis in *E. coli* and its application to the evolution of codon usage. PLOS ONE 7: e45635. 23029152 10.1371/journal.pone.0045635 PMC3461016

[34] Orth JD, Thiele I, Palsson BO (2010) What is flux balance analysis? Nat Biotechnol 28: 245–248. 20212490 10.1038/nbt.1614 PMC3108565

[35] Reed JL, Palsson BO (2003) Thirteen years of building constraint-based in silico models of *Escherichia coli* . J Bacteriol 185: 2692–2699. 12700248 10.1128/JB.185.9.2692-2699.2003 PMC154396

[36] Ibarra RU, Edwards JS, Palsson BO (2002) *Escherichia coli* K-12 undergoes adaptive evolution to achieve in silico predicted optimal growth. Nature 420: 186–189. 12432395 10.1038/nature01149

[37] Papini M, Nookaew I, Uhlen M, Nielsen J (2012) *Scheffersomyces stipitis*: a comparative systems biology study with the Crabtree positive yeast *Saccharomyces cerevisiae* . Microb Cell Fact 11: 136. 23043429 10.1186/1475-2859-11-136 PMC3528450

[38] Schellenberger J, Park JO, Conrad TM, Palsson BO (2010) BiGG: a Biochemical Genetic and Genomic knowledgebase of large scale metabolic reconstructions. BMC Bioinformatics 11: 213. 20426874 10.1186/1471-2105-11-213 PMC2874806

[39] Jensen PA, Lutz KA, Papin JA (2011) TIGER: Toolbox for integrating genome-scale metabolic models, expression data, and transcriptional regulatory networks. BMC Syst Biol 5: 147. 21943338 10.1186/1752-0509-5-147 PMC3224351

[40] Lomax RG (2007) Statistical Concepts: A Second Course. Hillsdale, NJ: Lawrence Erlbaum Associates.

[41] Breitling R, Armengaud P, Amtmann A, Herzyk P (2004) Rank products: a simple, yet powerful, new method to detect differentially regulated genes in replicated microarray experiments. FEBS Lett 573: 83–92. 15327980 10.1016/j.febslet.2004.07.055

