# Supplementary figures and images for: Prediction of Metabolic Flux Distribution from Gene Expression Data Based on the Flux Minimization Principle
Source: PLoS One. 2014 Nov 14;9(11):e112524. doi: 10.1371/journal.pone.0112524 (PMC4232356; doi:10.1371/journal.pone.0112524)

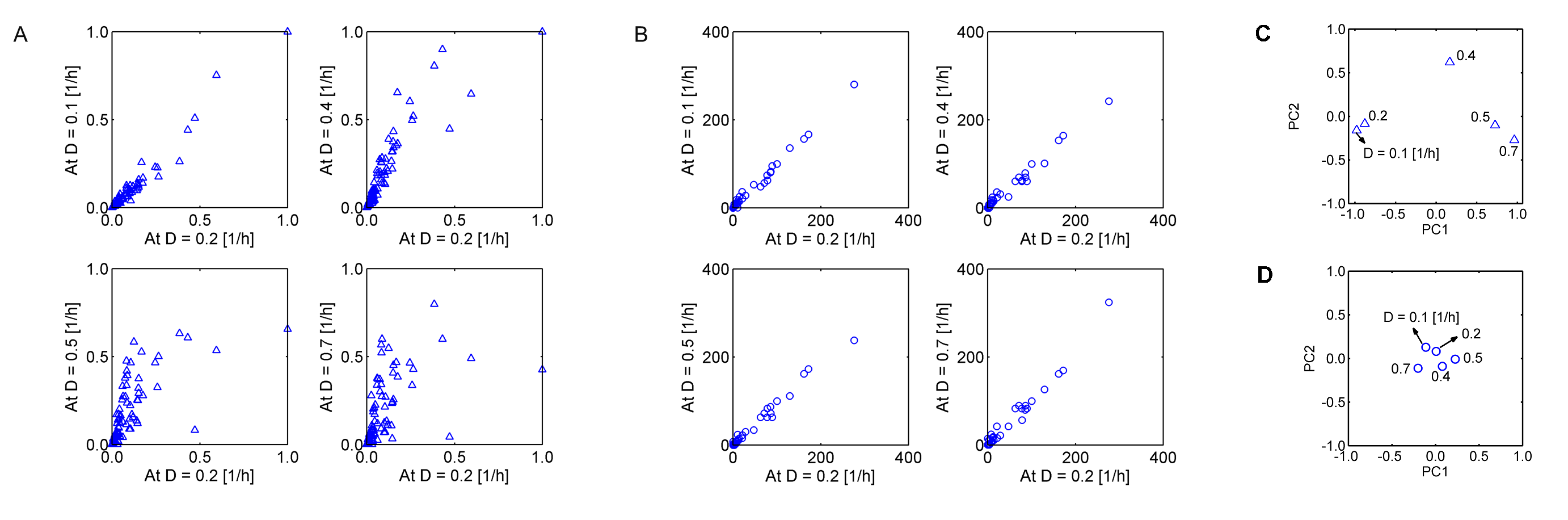

Supplement: Figure S1 — Wild-type Escherichia coli data collected at different dilution rates (Ishii et al., Science, 2007): gene expression data (triangles; A ), flux data (circles; B ), principal component analysis (PCA) using gene expression data (triangles; C ), and flux data (circles; D). Gene expression data were normalized to range from 0 to 1. Flux data were scaled so that the glucose uptake flux is 100 mmol/(gDW⋅h). For the PCA, both gene expression and flux data were normalized by their maximal value to range from 0 to 1. D denotes dilution rate [1/h]. (TIFF) [file pone.0112524.s001.tiff]
