# Supplementary material for: Prediction of Metabolic Flux Distribution from Gene Expression Data Based on the Flux Minimization Principle
Source: PLoS One. 2014 Nov 14;9(11):e112524. doi: 10.1371/journal.pone.0112524 (PMC4232356; doi:10.1371/journal.pone.0112524)
Supplement: Table S1 — Sum of squared error (SSE) of E-Fmin and other methods in predicting intracellular flux distributions in Saccharomyces cerevisiae at different glucose uptake rates, wild-type Escherichia coli at varied dilution rates, and mutated E. coli with single gene knockouts. (DOCX) [file pone.0112524.s002.docx]

**Supplementary Table S1. Sum of squared error (SSE)^*^ of E-Fmin and other methods in predicting intracellular flux distributions in wild-type** ***Escherichia coli* at varied dilution rates, mutated *E. coli* with single gene knockouts, and *Saccharomyces cerevisiae* at different uptake rates of glucose.**

| Strain | Perturbed condition | | E-Fmin | GIMME | FBA  (classical) | FBA  (flux min) | E-Flux | Lee et al. | iMAT  (outliers removed) |
| --- | --- | --- | --- | --- | --- | --- | --- | --- | --- |
| *S. cerevisiae* | Uptake flux of  glucose,  mmol/(gDW⋅h) | 16.5 | 1.9 | 26.1 | 26.8 | 21.7 | 0.4 | 0.5 | 33.7 |
|  |  | 11 | 0.8 | 6.4 | 8.6 | 7.0 | 1.2 | 2.4 | 11.2 |
|  |  | **Average** | **1.4** | **16.3** | **17.7** | **14.4** | **0.8** | **1.4** | **22.5** |
| Wild-type  *E. coli* | Dilution  rate, 1/h | 0.1 | 61.1 | 86.2 | > 10^3^ | 47.2 | 376 | 401 | 67.3 |
|  |  | 0.2 | 77.4 | 92.9 | > 10^3^ | 50.0 | 399 | 380 | 81.7 |
|  |  | 0.4 | 45.7 | 51.1 | > 10^3^ | 17.6 | 249 | 342 | 44.9 |
|  |  | 0.5 | 35.1 | 75.9 | > 10^3^ | 31.6 | 174 | 362 | 76.2 |
|  |  | 0.7 | 69.5 | 119 | > 10^3^ | 63.5 | 236 | 542 | 68.0 |
|  |  | **Average** | **57.8** | **85.0** | **> 10^3^** | **42.0** | **287** | **405** | **67.6** |
| Mutant  *E. coli* | Single  gene  disruption | galM | 72.5 | 99.5 | > 10^3^ | 57.1 | 411 | 402 | 75.7 |
|  |  | glk | 113 | 130 | > 10^3^ | 76.9 | 395 | 404 | 106 |
|  |  | pgm | 93.6 | 119 | > 10^3^ | 63.1 | 281 | 396 | 89.3 |
|  |  | pgi | 36.3 | 76.6 | > 10^3^ | 48.9 | 287 | 397 | 34.9 |
|  |  | pfkA | 76.7 | 83.7 | > 10^3^ | 43.4 | 291 | 347 | 83.9 |
|  |  | pfkB | 46.8 | 71.8 | > 10^3^ | 37.3 | 305 | 399 | 51 |
|  |  | fbp | 72.9 | 90.2 | > 10^3^ | 54.9 | 365 | 364 | 96.7 |
|  |  | fbaB | 64.4 | 77.8 | > 10^3^ | 38.7 | 356 | 369 | 70 |
|  |  | gapC | 66.2 | 94.2 | > 10^3^ | 54.6 | 365 | 397 | 73.5 |
|  |  | gpmA | 74.1 | 90.4 | > 10^3^ | 40.7 | 337 | 346 | 80.3 |
|  |  | gpmB | 128 | 148 | > 10^3^ | 91.7 | 412 | 424 | 117 |
|  |  | pykA | 69.3 | 86.3 | > 10^3^ | 46.2 | 339 | 406 | 77 |
|  |  | pykF | 38.9 | 57.7 | > 10^3^ | 35 | 324 | 405 | 45.5 |
|  |  | ppsA | 81.7 | 94.9 | > 10^3^ | 47.2 | 408 | 391 | 66.2 |
|  |  | zwf | 119 | 86.3 | > 10^3^ | 94.8 | 401 | 410 | 122 |
|  |  | pgl | 82.4 | 99.6 | > 10^3^ | 55.1 | 291 | 389 | 85.3 |
|  |  | gnd | 136 | 99.9 | > 10^3^ | 105 | 451 | 428 | 127 |
|  |  | rpe | 123 | 173 | > 10^3^ | 115 | 396 | 467 | 137 |
|  |  | rpiA | 98.9 | 132 | > 10^3^ | 77.3 | 288 | 462 | 98.6 |
|  |  | rpiB | 135 | 170 | > 10^3^ | 109 | 468 | 526 | 102 |
|  |  | tktA | 193 | 226 | > 10^3^ | 154 | 549 | 533 | 158 |
|  |  | tktB | 102 | 112 | > 10^3^ | 61.9 | 391 | 383 | 94.0 |
|  |  | talA | 87.6 | 94.2 | > 10^3^ | 49.4 | 359 | 361 | 86.5 |
|  |  | talB | 98.6 | 108 | > 10^3^ | 59.5 | 425 | 386 | 92.8 |
|  |  | **Average** | **92.1** | **109.2** | **> 10^3^** | **67.4** | **371** | **408** | **90.4** |

^*^$SSE\equiv\frac{\sum_{i} \left(r_{i}-r_{\exp,i}\right) ^{2}}{\left(\sum_{i} r_{\exp,i}^{2}\right)^{1/2}}$, where *r*_exp,_*_i_* denotes individual experimental data and *r_i_* denotes the model estimation of *r*_exp,_*_i_*.
