# Supplementary material for: Prediction of Metabolic Flux Distribution from Gene Expression Data Based on the Flux Minimization Principle
Source: PLoS One. 2014 Nov 14;9(11):e112524. doi: 10.1371/journal.pone.0112524 (PMC4232356; doi:10.1371/journal.pone.0112524)
Supplement: Table S2 — Comparison of exchange fluxes of Saccharomyces cerevisiae between experimental data and computational predictions. (DOCX) [file pone.0112524.s003.docx]

**Supplementary Table S2.** **Comparison of exchange fluxes of *Saccharomyces cerevisiae* between experimental data and computational predictions.**

| **Uptake rate of glucose = 16.5** **mmol/(gDW·h)** | | | | | | | | | | |
| --- | --- | --- | --- | --- | --- | --- | --- | --- | --- | --- |
|  | Exp. | E-Fmin | GIMME | FBA  (classical) | | FBA  (flux min) | | E-Flux | Lee | iMAT |
| Ethanol | 23.8* | 27.3 | 0 | 0 | | 0 | | 23.7 | 25.7 | 0 |
| CO_2_ | 22.7 | 29.4 | 42.3 | 42.9 | | 37.6 | | 25.2 | 23.1 | 48.5 |
| Glycerol | 3.54 | 0 | 0 | 0 | | 0 | | 0.97 | 0 | 0 |
| Acetate | 0.31 | 0 | 0 | 0 | | 0 | | 0 | 0.016 | 0 |
| Trehalose | 0.036 | 0 | 0 | 0 | | 0 | | 0 | 0.030 | 0 |
| Lactate | 0.009 | 0 | 0 | 0 | | 0 | | 0 | 0.030 | 0 |
| Biomass | 0.36 | 0.46 | 1.55 | 1.72 | | 1.72 | | 0.33 | 0 | 1.55 |
| **Uptake rate of glucose = 11.0 mmol/(gDW****·h)** | | | | | | | | | | |
|  | Exp. | E-Fmin | GIMME | | FBA  (classical) | | FBA  (flux min) | E-Flux | Lee | iMAT |
| Ethanol | 13 | 17 | 0.20 | | 0 | | 0 | 16.4 | 16.2 | 0 |
| CO_2_ | 21 | 19.5 | 18.7 | | 28.6 | | 25 | 16.9 | 13.9 | 32.3 |
| Glycerol | 2.17 | 0 | 0 | | 0 | | 0 | 0.55 | 0.13 | 0 |
| Acetate | 0.24 | 0 | 0 | | 0 | | 0 | 0 | 0.009 | 0 |
| Trehalose | 0.023 | 0 | 0 | | 0 | | 0 | 0 | 0.022 | 0 |
| Lactate | 0.006 | 0 | 0 | | 0 | | 0 | 0 | 0.018 | 0 |
| Biomass | 0.22 | 0.31 | 1.03 | | 1.2 | | 1.2 | 0.22 | 0 | 1.03 |

*Flux units: mmol/(gDW·h) for metabolites and 1/h for biomass.
