# Supplementary material for: Prediction of Metabolic Flux Distribution from Gene Expression Data Based on the Flux Minimization Principle
Source: PLoS One. 2014 Nov 14;9(11):e112524. doi: 10.1371/journal.pone.0112524 (PMC4232356; doi:10.1371/journal.pone.0112524)
Supplement: Table S3 — Comparison of intracellular fluxes of wild-type Escherichia coli at a dilution rate (D) of 0.1 (1/h) between experimental data and computational predictions. (DOCX) [file pone.0112524.s004.docx]

**Supplemental Table S3. Comparison of intracellular fluxes of wild-type *Escherichia coli* at the dilution rate (D) of 0.1 (1/h) between experimental data and computational predictions.**

|  | Exp | E-Fmin | GIMME | FBA  (classical) | FBA (flux min) | E-Flux | Lee | iMAT |
| --- | --- | --- | --- | --- | --- | --- | --- | --- |
| Glucose+PEP → G6P+PYR | 100* | 100 | 100 | 100 | 100 | 0.79 | 100 | 100 |
| G6P → F6P | 62.2 | 33.4 | 36.5 | 48.6 | 48.6 | 0.79 | 99.4 | 4.78 |
| F6P → F1,6P | 80.2 | 41.5 | 71.3 | 74.5 | 67.5 | 8.29 | 99.4 | 60.8 |
| F1,6P → DHAP+G3P | 80.2 | 41.5 | 71.3 | 74.5 | 67.5 | 8.29 | 99.4 | 60.8 |
| DHAP → G3P | 80.2 | 68.3 | 70.1 | 73.2 | 73.2 | 3.85 | 99.4 | 59.5 |
| G3P → 3PG | 167 | 155 | 158 | 160 | 160 | 24 | 199 | 148 |
| 3PG → PEP | 157 | 139 | 144 | 143 | 143 | 19 | 199 | 133 |
| PEP → PYR | 52.9 | 0 | 23.4 | 7.03 | 0 | 0 | 50.1 | 0 |
| PYR → AcCoA+CO2 | 136 | 101 | 97.8 | 79.7 | 79.7 | 17.2 | 0 | 75.4 |
| G6P → 6PG | 36.3 | 66.6 | 63.5 | 51.4 | 51.4 | 0 | 0.63 | 95.2 |
| 6PG → Ru5P+CO2 | 36.3 | 66.6 | 63.5 | 51.4 | 51.4 | 1.24 | 0 | 95.2 |
| Ru5P → X5P | 18.7 | 37.1 | 35.6 | 26.8 | 26.8 | -3.81 | 0 | 56.8 |
| Ru5P → R5P | 17.6 | 29.1 | 27.5 | 24.2 | 24.2 | 4.92 | 0 | 38.1 |
| R5P+X5P → S7P+G3P | 10.8 | 20.3 | 19.4 | 15.2 | 15.2 | -1.34 | 0 | 30 |
| S7P+G3P → E4P+F6P | 10.8 | 20.3 | 19.4 | 15.2 | 15.2 | -1.34 | 0 | 30 |
| X5P+E4P → F6P+G3P | 7.8 | 16.8 | 16.2 | 11.6 | 11.6 | -2.47 | 0 | 26.8 |
| AcCoA+OAA → CIT | 82.2 | 39.9 | 21.6 | 48.1 | 48.1 | 7.21 | 0 | 47 |
| CIT → ICT | 82.2 | 39.9 | 21.6 | 48.1 | 48.1 | 7.21 | 0 | 47 |
| ICT → 2-KG+CO2 | 56.4 | 15.1 | 9.32 | 48.1 | 48.1 | 3.26 | 0 | 47 |
| 2-KG → SUC+CO2 | 48.1 | 0 | -4.55 | 1×10^5^ | 32.7 | -1.59 | 0 | 1×10^5^ |
| SUC → FUM | 73.9 | 29.8 | 12.3 | 37.8 | 37.8 | 3.94 | 0 | 37.7 |
| FUM → MAL | 73.9 | 39.6 | 21.3 | 47.8 | 47.8 | 35.7 | 0 | 46.7 |
| MAL → OAA | 95.5 | 64.4 | 33.7 | 47.8 | 47.8 | 39.6 | -24.4 | 46.7 |
| PEP+CO2 → OAA | 0.2 | 3.22 | 13.3 | 28.5 | 28.5 | 5.04 | 49.3 | 25.6 |
| MAL → PYR+CO2 | 4.1 | 0 | 0 | 0 | 0 | 0 | 24.4 | 0 |
| ICT → Glyoxylate+SUC | 25.7 | 24.8 | 12.3 | 0 | 0 | 3.94 | 0 | 0 |
| Glyoxylate+AcCoA → MAL | 25.7 | 24.8 | 12.3 | 0.01 | 0.01 | 3.94 | 0 | 0.01 |
| AcCoA → Acetate | 0 | 0 | 31.4 | -5.59 | -5.59 | -1.76 | 31.4 | -5.04 |
| PYR → Lactate | 0 | 0 | 0 | 0 | 0 | -14.7 | 200 | 0 |
| AcCoA → Ethanol | 0 | 0 | 0 | 0 | 0 | 0 | 0 | 0 |
| G6P → (Cell synthesis) | 1.5 | 1.94 | 1.78 | 1.97 | 1.97 | 0.62 | 0 | 1.78 |
| F6P → (Cell synthesis) | 0.7 | 0.67 | 0.61 | 0.68 | 0.68 | 0.22 | 0 | 0.61 |
| R5P → (Cell synthesis) | 6.8 | 8.49 | 7.78 | 8.64 | 8.64 | 2.72 | 0 | 7.78 |
| E4P → (Cell synthesis) | 3 | 3.42 | 3.13 | 3.48 | 3.48 | 1.1 | 0 | 3.13 |
| G3P → (Cell synthesis) | 1.1 | 1.22 | 1.12 | 1.24 | 1.24 | 0.39 | 0 | 1.12 |
| 3PG → (Cell synthesis) | 10.2 | 14.2 | 13 | 14.4 | 14.4 | 4.54 | 0 | 13 |
| PEP → (Cell synthesis) | 3.7 | 4.91 | 4.5 | 5 | 5 | 1.58 | 0 | 4.5 |
| PYR → (Cell synthesis) | 21 | 26.8 | 24.6 | 27.3 | 27.3 | 8.6 | 0 | 24.6 |
| AcCoA → (Cell synthesis) | 28.1 | 35.5 | 32.5 | 36.1 | 36.1 | 11.4 | 0 | 32.5 |
| OAA → (Cell synthesis) | 13.6 | 16.9 | 15.5 | 17.2 | 17.2 | 5.42 | 0 | 15.5 |
| 2KG → (Cell synthesis) | 8.3 | 39 | 35.7 | 39.7 | 39.7 | 12.5 | 0 | 35.7 |
| CO2 → (Evolution) | 281 | 202 | 172 | 206 | 206 | 22 | 0 | 245 |

* Fluxes were normalized such that the uptake rate of glucose became 100 mmol/(gDW·h).
